# Physically Consistent Whole-Body Kinematics Assessment Based on an RGB-D Sensor. Application to Simple Rehabilitation Exercises

**DOI:** 10.3390/s20102848

**Published:** 2020-05-17

**Authors:** Jessica Colombel, Vincent Bonnet, David Daney, Raphael Dumas, Antoine Seilles, François Charpillet

**Affiliations:** 1 Université de Lorraine, CNRS, Inria, LORIA, F-54000 Nancy, France; jessica.colombel@inria.fr (J.C.); francois.charpillet@inria.fr (F.C.); 2 LISSI, Univ. Paris Est Creteil, F-94400 Vitry, France; 3 Inria Bordeaux Sud Ouest - IMS (UMR 5218), F-33405 Talence, France; david.daney@inria.fr; 4 Univ. Lyon, Univ. Gustave Eiffel, LBMC (UMR T9406), F-69675 Lyon, France; raphael.dumas@univ-eiffel.fr; 5 NaturalPad, 34090 Montpellier, France; antoine@naturalpad.fr

**Keywords:** markerless human motion analysis, extended Kalman filter and rehabilitation

## Abstract

This work proposes to improve the accuracy of joint angle estimates obtained from an RGB-D sensor. It is based on a constrained extended Kalman Filter that tracks inputted measured joint centers. Since the proposed approach uses a biomechanical model, it allows physically consistent constrained joint angles and constant segment lengths to be obtained. A practical method that is not sensor-specific for the optimal tuning of the extended Kalman filter covariance matrices is provided. It uses reference data obtained from a stereophotogrammetric system but it has to be tuned only once since it is task-specific only. The improvement of the optimal tuning over classical methods in setting the covariance matrices is shown with a statistical parametric mapping analysis. The proposed approach was tested with six healthy subjects who performed four rehabilitation tasks. The accuracy of joint angle estimates was assessed with a reference stereophotogrammetric system. Even if some joint angles, such as the internal/external rotations, were not well estimated, the proposed optimized algorithm reached a satisfactory average root mean square difference of 9.7${}^{\circ }$ and a correlation coefficient of 0.8 for all joints. Our results show that an affordable RGB-D sensor can be used for simple in-home rehabilitation when using a constrained biomechanical model.

## 1. Introduction

Physical rehabilitation is of crucial importance to maintaining or restoring body movement and function. To improve its effectiveness, a rehabilitation session usually requires the presence of a physiotherapist and/or a clinician to assess, encourage, and correct the patient in their realization of the prescribed physical exercises. For example, squat and walking exercises are increasingly used in the rehabilitation process and mobility assessment of age-related lower-limb pathologies. Having a reliable estimate of a patient’s joint angles during the rehabilitation process is of great importance for mobility assessment to better understand the physical and functional evolution of the patient’s recovery. However, making a quantitative assessment of whole-body motions from a visual inspection is not an easy task, and there will be large inter/intra-clinician variability in the motion evaluation [1]. It has been reported that therapists tend to underestimate the range of motion by 9${}^{\circ }$ on average [2]. Measurement tools which are capable of providing a consistent quantitative analysis are of crucial importance for the clinical community. Nevertheless, even when using these measurement tools, typical errors with kinematics obtained from inertial measurement units (IMU) or reference stereophotogrammetric systems (SS) can reach 22${}^{\circ }$ for knee rotations and 10${}^{\circ }$ for shoulder rotations [3]. This is due more to experimental and model reconstruction errors than to the sensor itself. Kinematics data, if provided in real-time, can also be integrated as feedback in a game to increase patient interest and motivation [4]. Usually reference kinematics is estimated using a SS that is accurate but costly and requires a rather complex hardware installation, making its use restricted to laboratory settings. Consequently, such a system cannot be used for in-home applications. Recently, numerous affordable and easy-to-use systems based on IMU [5], RGB cameras [6], and RGB-D sensors [7] have been developed to generalize human motion analysis. Each of these systems has some drawbacks (e.g., calibration, occlusion, accuracy), but it is often possible to improve their accuracy for human motion analysis in different ways. The main advantage of the markerless camera-based system is that they do not rely on embedded sensors or markers and do not require any particular dressing or undressing action. In this context, the next section of this paper will focus on the literature related to camera-based systems.

## 2. Background

### 2.1. Related Work

Recently, markerless visual motion capture based on an RGB camera and a machine learning algorithm has been used to estimate human motion in challenging scenarios [8]. However, such approaches are not yet real-time or accurate enough to reliably estimate a patient’s joint angles and thus are not accessible to the clinical community. Progress is expected in terms of accuracy thanks to new methods that combine machine learning and model-based approaches [9]. Devices based on RGB-depth sensors with embedded skeleton tracking algorithms are already available to the wider public. The most well-known of these is the Kinect v1 sensor (K1S); however, this was shown to be insufficiently accurate for rehabilitation assessment due to segment length variations [10]. With the release of the Kinect v2 sensor (K2S) in 2014, new studies [11,12] showed that joint center position (JCP) estimates have significantly improved when compared to those estimated by the K1S. In addition, fewer outliers were observed as K2S is more robust to occlusions and body rotations thanks to a more accurate depth map. Naeemabadi et al. [12] compared K2S and K1S JCP estimates with those obtained with an SS in the context of knee rehabilitation. They exhibited a promising average root mean square difference (RMSD) of 4.9${}^{\circ }$ for K2S, whereas the RMSD for K1S was much higher at 13.4${}^{\circ }$. Wochatz et al. [13] explored the validity of using a K2S during squat, hip abduction and lunge rehabilitation exercises. They concluded that the accuracy decreased with small movement amplitudes and with the increase of movement complexity. This was later confirmed by Galna et al. [14], who stated that the Kinect can accurately measure the timing and gross spatial characteristics of clinically relevant movements but not with the same spatial accuracy for smaller movements. With a K2S, spatio-temporal parameters such as walking speed, time of movement up and down or the mean sway velocity of the body can be estimated accurately [15,16]. However, it was shown that, since the segment lengths were not constant in time, the resultant joint angles were not reliable enough for gait analysis [15]. For upper limb rehabilitation, Kuster et al. [17] showed an average RMSD of 7${}^{\circ }$ for the trunk and 11.8${}^{\circ }$ for shoulder flexion/extension when compared to an SS. This was considered to be sufficiently accurate for the investigated tasks. These studies seem to demonstrate that the K2S may be used for quantitative rehabilitation assessment if data from the skeleton extractor were more consistent.

Numerous methods have been developed in the literature to improve kinematic estimates of RGB-D-like sensors. Three different approaches, which can be combined, are usually proposed to improve the accuracy of JCP estimates: (i) improvement of the skeleton tracking algorithm, (ii) adding sensors and (iii) filtering.

Regarding the improvement of the skeleton tracking algorithm, Han et al. [18], in a survey on skeleton tracking algorithms, showed that the main drawback of artificial intelligence approaches was their computational expense, which means that they are not suitable for real-time applications. Model-based methods are considered suitable for real-time applications but suffer from cumbersome and sensitive parameter tuning. Plantard et al. [19] designed a pose graph filter that identified the most probable pose in a database constructed a priori. Once a correspondence was found, the measured poses were reconstructed, increasing their robustness to occlusion and overall accuracy. The RMSD between the reconstructed pose and the reference system was 0.09 m for the estimated 3D joint center positions. This is better than the K2S output, which was estimated at 0.15 m.

Regarding the addition of sensors, Kim et al. [20] proposed the determination of JCP with multiple K2S devices. The merging of several sets of skeleton data allowed the authors to reduce the number of occlusions. They compared their approach with the output of a commercial IMU system and reached 80% motion similarity while a single K2S obtained only 20% similarity. Dual K1S devices were also used in combination with a musculoskeletal model [21]. This setup showed encouraging results in the estimation of the shoulder abduction/adduction angle (correlation up to 0.89). However, it was found to not be sufficiently accurate to estimate lower-limb motions, as the tracking of the hip internal/external rotation angles presented a correlation of −0.63. Sensor fusion was also proposed to improve the accuracy of joint kinematics. In a survey [22], it was suggested that merging Kinect and IMU data can prevent occlusions and improve joint angle estimate accuracy [23,24]. However, using embedded sensors is undesirable for in-home rehabilitation.

Finally, regarding filtering, Tripathy et al. [25] developed a genetic algorithm based on a particle filtering method to constrain the length of segments during shoulder motions. The mean absolute difference between their segment length estimates and the reference estimates was only 3.44%. Unfortunately, they did not consider the use of this algorithm for joint angle estimates. Shu et al. [26] used an extended Kalman filter (EKF) to improve kinematic estimates from pose data. Unfortunately, their algorithm was only implemented with the head joints, but they were able to obtain an accuracy of 0.039 m on the head JCP with Kalman filtering. Skals et al. [21] used multibody kinematic optimization (MKO) instead of an EKF to introduce kinematic constraints. Both methods seem to provide similar results, but the MKO cannot be implemented in real-time. This shows the importance of using anthropomorphic constraints, a kinematic model and temporal relations between the state variables to improve the accuracy of kinematics estimates from Kinect data. Moreover, model-based approaches have the advantages of not being sensor-specific and requiring very little training/testing data compared to the methods based on machine learning.

### 2.2. Contribution

The K2S was designed for in-home applications; however, its data, which are not consistent and thus lead to variable segment lengths and infeasible joint angle estimates, should be corrected for rehabilitation assessment. This paper proposes the use of a new constrained extended Kalman filter (CEKF) based on a whole-body model of the human locomotor system including anthropomorphic constraints to improve the accuracy of joint angle estimates from K2S data. The contributions of this paper are as follows:

- A new CEKF is proposed to obtain physically consistent joint angles in real-time and at low computational cost. The constraints impose fixed segment lengths which do not require a subject- specific calibration and joint angle physiological limits;
- A pragmatic method is proposed to optimize the measurement and process covariance matrices of the CEKF based on the SS data, depending only on the investigated task and not on the subject. Thus, as for the segment lengths, all model and method calibrations can be performed a priori without involving the subjects under study;
- An experimental validation based on a joint angle accuracy analysis (i.e., CEKF vs. MKO) of the whole-body is presented.

## 3. Materials and Methods

### 3.1. Mechanical Model

**Figure 1:**
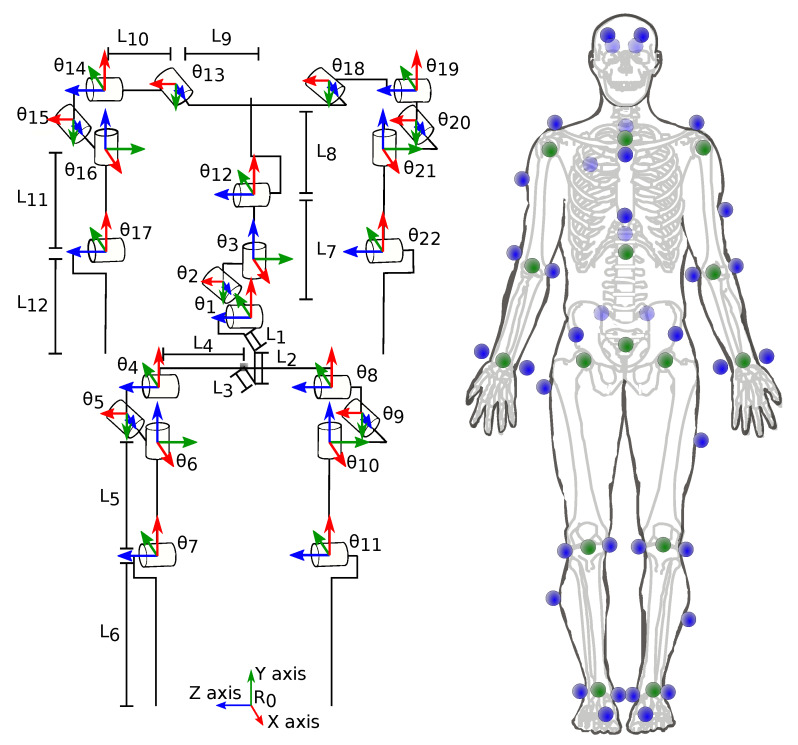
(**Left**) A 22 degree-of-freedom model of the locomotor system. $\theta_{i}$ is the *i*th joint angle corresponding to a rotation along the *i*th local Z axis and $L_{j}$ is the length of the segment *j*. (**Right**) Location of the retro-reflective markers (blue), used by the reference stereophotogrammetric system (SS), and of the Kinect sensor (K2S)-estimated joint center position (JCP) (green).

The mechanical model is used to relate the output of the K2S with the human joint kinematics through the calculation of the forward kinematics model. Figure 1 shows the proposed mechanical model composed of $N_{\theta }=22$ revolute joints (θ) and $N_{L}=12$ segments. The green dots in this figure represent the $N_{J}=15$ retained JCPs estimated by the K2S. The forward kinematics model was calculated to express the position of each estimated JCP as a function of θ ($N_{\theta }\times 1$) and of segment lengths L ($N_{L}\times 1$) using the modified Denavit–Hartenberg convention [27]. Because of this convention, all *Z-axes* of the model correspond to an actual rotation. Nevertheless, the joint definition follows the recommendations of the International Society of Biomechanics [28,29].

### 3.2. Constrained Extended Kalman Filter

The aim of the proposed CEKF is to estimate the state vector $X_{k}={\left[\theta \;\dot{\theta }\;\ddot{\theta }\;L\right]}^{T}$ while tracking the 3D JCP T ($(3N_{J})\times 1$) provided by the K2S. $\dot{\theta }$ and $\ddot{\theta }$ are the $N_{\theta }\times 1$ vectors of joint velocities and accelerations, respectively. The state and measurement vectors are modeled as follows at time *k*:

(1) $$\begin{array}{cc} X_{k} & =f\left(X_{k-1}\right)+w_{k-1}\\ Z_{k} & =h\left(X_{k}\right)+v_{k} \end{array}$$

where *f* is the state model described by Equation (2), *h* is the measurement model given by the forward kinematics model from the mechanical model described in Section 3.1, $w_{k}$ represents the system noise, defined by p(w)∼N(0,Q) with Q being the model covariance noise matrix, and $v_{k}$ represents the measurements noise defined by p(v)∼N(0,R), with R ($3N_{J}\times 3N_{J}$) being the covariance matrix of measurement noise.

The proposed state model *f* is approximated by a linear form, denoted as F. It is assumed that the joint angles and velocities evolve linearly and that the joint accelerations and segment lengths are constant:

(2) $$\begin{array}{cc} F & =\left[\begin{array}{cc} \left[\begin{array}{ccc} I_{N_{\theta }\times N_{\theta }} & \Delta tI_{N_{\theta }\times N_{\theta }} & \frac{\Delta t^{2}}{2}I_{N_{\theta }\times N_{\theta }}\\ 0_{N_{\theta }\times N_{\theta }} & I_{N_{\theta }\times N_{\theta }} & \Delta tI_{N_{\theta }\times N_{\theta }}\\ 0_{N_{\theta }\times N_{\theta }} & 0_{N_{\theta }\times N_{\theta }} & I_{N_{\theta }\times N_{\theta }} \end{array}\right] & 0_{3N_{\theta }\times N_{L}}\\ 0_{N_{L}\times 3N_{\theta }} & I_{N_{L}\times N_{L}} \end{array}\right] \end{array}$$

where Δt is the sample time and 0 and I are the null and identity matrices, respectively.

At each time step, the prediction of the a priori state vector is calculated as follows:

(3) $$\begin{array}{cc} {\hat{X}}_{k}^{-} & =F{\hat{X}}_{k-1}\\ P_{k}^{-} & =FP_{k-1}F^{T}+Q \end{array}$$

where $P_{k}^{-}$ is the a priori estimation of the error co-variance matrix.

In order to ensure the physical consistency of the state vector estimate, inequality constraints are added to the EKF for each joint angle and for segment lengths. Gupta et al. [29] presented a method to easily implement constraints on the state vector estimate as follows by calculating a restricted Kalman gain matrix $K_{k}^{R}$:

(4) $$\begin{array}{cc} C{\hat{X}}_{k} & \leq d \end{array}$$

where $d={\left[\theta^{-}\;\theta^{+}\;L^{-}\;L^{+}\right]}^{T}$ and C are the inequality constraint vectors and matrices, respectively.

$\theta^{-}$ and $\theta^{+}$ are the maximal and minimal joint angle values taken from the literature, and $L^{-}$, $L^{+}$ were set to ±20% of their initial estimate.

The update of the restricted state vector can then be calculated based on the new measurement vector as follows:

(5) $$\begin{array}{cc} S_{k} & =H_{k}P_{k}^{-}H_{k}^{T}+R\\ K_{k}^{R} & =K_{k}-C^{T}{\left(CC^{T}\right)}^{-1}\left(C{\hat{X}}_{k}^{-}-d\right){\left(v_{k}^{T}S_{k}^{-1}v_{k}\right)}^{-1}v_{k}^{T}S_{k}^{-1}\\ {\hat{X}}_{k} & ={\hat{X}}_{k}^{-}+K_{k}^{R}\left(Z_{k}-h\left({\hat{X}}_{k}^{-}\right)\right)\\ P_{k} & =\left(I-K_{k}^{R}H_{k}\right)P_{k}^{-} \end{array}$$

where $H_{k}$ is the Jacobian matrix $\frac{\partial h}{\partial X_{k}}$, $S_{k}$ is the innovations co-variance matrix, and $P_{k}$ is the error co-variance matrix.

Note that the segment lengths are also forced to converge to a constant value (see the definition of F and Q) because physical lengths are considered constant in the human body.

### 3.3. Participants and Procedures

**Figure 2:**
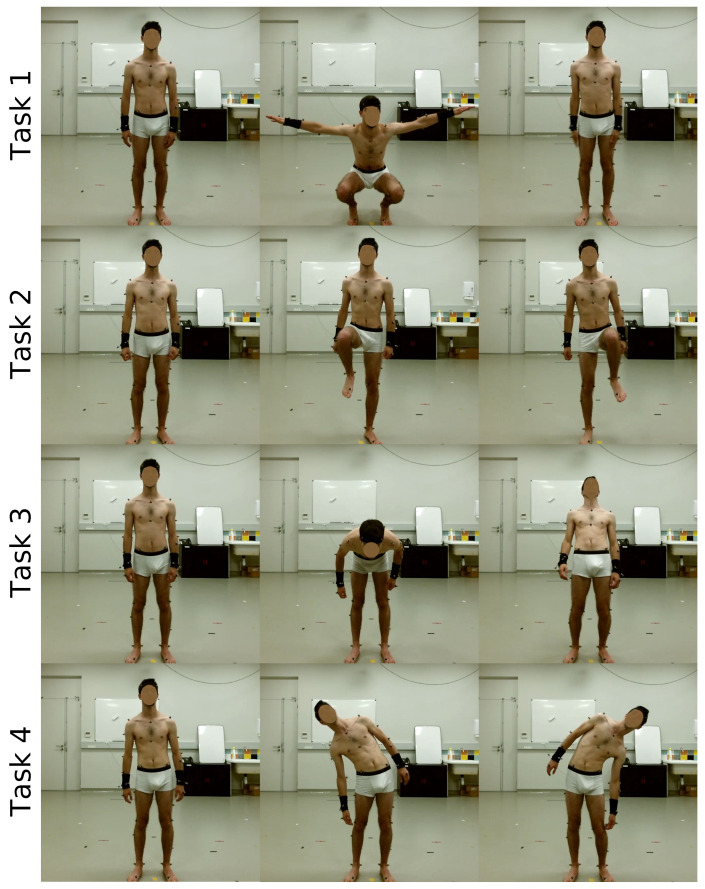
Tasks for measurement: (**1**) squat with lateral arm extensions, (**2**) stepping (left and right), (**3**) body tilt in sagittal plane and (**4**) body tilt in frontal plane.

This study followed the principles of the Declaration of Helsinki. Six healthy male participants (age: 32±17 years; weight: 78±9 kg; height: 1.77±0.05 m) participated in the experiment after giving their written informed consent and permission for their image to be used. Participants performed four tasks (illustrated in Figure 2): a deep squat with lateral extensions of their arms, stepping in position with their left and right legs, tilting their trunk in their sagittal plane and tilting their trunk in frontal planes. Each participant performed ten consecutive repetitions for each movement. All trials started in a resting position: standing, arms along the body, facing the K2S.

Whole-body motion was simultaneously collected by a reference SS consisting of eight infrared cameras (VICON, MX-16, Oxford metrics, 100 Hz) and by one Microsoft K2S (30 Hz). The skeletal tracking method implemented in Kinect SDK v2.0 was used even if its functioning principle has not been fully disclosed by Microsoft [30]. For an optimal field of view, the K2S was placed in front of the participant at 2.5 m and at 0.85 m above the floor, as recommended in the literature [30]. Moreover, the combined use of K2S and SS, both of which are infra-red sensors, might degrade the quality of K2S data [31]. Thus, the SS was not located in the direct field of view of the K2S and the participants were always less than 3 m away from the K2S. The SS reflective markers could also impact the estimate of K2S JCP, and thus their number was minimized and their position was as far as possible from the human joint centers. Consequently, to estimate the reference joint angles, 39 reflective markers based on the popular reduced marker-set “Plug-In Gait” [32] were used here (Figure 1). The trajectory of these reflective markers was fed to a state-of-the-art MKO [3] to obtain the reference joint trajectories. Note that, without this optimization step, the segment lengths may have been non-constant in time with the SS.

### 3.4. Cekf Parameter Adjustment

**Figure 3:**
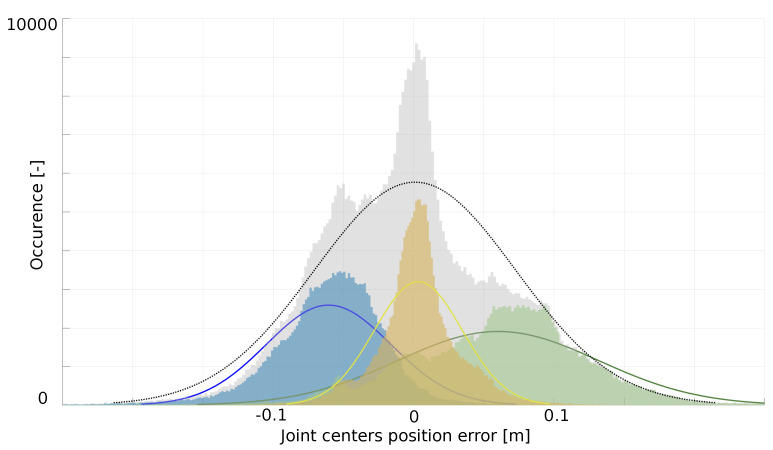
Distribution of the difference between joint position centers estimated from the SS and from the K2S obtained for all recorded data along the x (blue), y (green) and z (yellow) axes. The grey distribution indicates the average one.

The convergence rate and stability of the EKF filter depend on the tuning of its parameters; i.e., the process and measurement covariance matrices, Q and R, the initialization of the error covariance matrix $P_{0}$ and the initial value of state variables. The tuning of these parameters is rarely discussed in the human motion analysis literature. However, there is a consensus in the methodology for the tuning of R based on the fact that the measurement noise is supposedly Gaussian-distributed. This hypothesis is validated by calculating the difference between the SS’s JCP estimate and that of the K2S. Figure 3 shows the Gaussian-like distribution of the average measurement noise for all JCPs for each axis. The Gaussian-distributed error of the x-axis has a mean μ=−0.06 m and variance $\sigma^{2}=0.0019$; for the y-axis, these values are μ=0.06 and $\sigma^{2}=0.0050$; and for the z-axis, these values are μ=−0.003 and $\sigma^{2}=0.0009$. Finally, for the averaged distribution (i.e., for all dimensions), we obtain μ=0.00 and $\sigma^{2}=0.0052$.

The initial values of the state vector were set using another MKO [3] with the aim of finding the joint angles and segment lengths that fit the K2S’s JCP estimate over the first sample as well as possible. To give the same influence to all joints, $P_{0}$ was set to the identity matrix. The tuning of the elements of Q was sensitive and was task and joint-dependent [33]. The first set of state variables that were supposed to be constant and independent during the task were the segment lengths. To enforce their convergence to a constant value, their corresponding elements in Q were set to zero. This guaranteed the convergence of the corresponding error covariance P to zero. For the tuning of Q elements corresponding to the joint positions, velocities and accelerations, two tuning methods were investigated: the first one, based on [33,34], assumed a priori knowledge of the state variable frequency content; the second one was based on a task-specific optimization process using the reference joint angles obtained from the SS.

#### 3.4.1. Data-Driven Tuning of Matrix Q

In human motion analysis, the process noise covariance matrix can be tuned with a priori knowledge of the frequency content of the investigated motion. As proposed by De Groote et al. [34], this is calculated for each joint *k* as follows:

(6) $$\begin{array}{c} Q_{j}=\sigma_{k}^{2}G^{T}G\;\mathrm{where}:\;G=\left[\begin{array}{ccc} \frac{\Delta t^{3}}{3!} & \frac{\Delta t^{2}}{2} & \Delta t \end{array}\right] \end{array}$$

where $\sigma_{k}^{2}$ is the noise covariance factor, defined as follows:

(7) $$\begin{array}{c} \sigma_{k}^{2}=\frac{{\left(\left(A\omega_{max}^{4}e^{\omega_{max}\Delta t}/4\pi \right)\Delta t\right)}^{2}}{3\Delta t}. \end{array}$$

where $\omega_{max}$ is the cutoff frequency and *A* is the joint trajectory amplitude. The estimations of $\omega_{max}$ and *A* are obtained thanks to a fast Fourier transform on the joint trajectories obtained from the SS and MKO for each task.

#### 3.4.2. Optimal Tuning of Q Matrix

An optimization process with the aim of determining the elements of Q, which minimizes the square difference between the estimated joint angles and the reference ones obtained from the SS and MKO, was developed. When dealing with an EKF for motion analysis, the hypothesis is that the tuning of Q is task-dependent [34]. Consequently, the data of one randomly chosen subject were used to identify the elements of Q. The same identified values were then used for the other subjects. Since the optimization process is computationally expensive, due to the fact that the EKF will have to be run several thousand times, and because the investigated tasks are symmetric, it was proposed to use the same set of values for left and right joints. A total of 11 parameters, with four parameters for the legs, four parameters for the arms, two parameters for the base-link and one for the trunk, needed to be identified. The elements of R were set from the experimental distribution analysis described in Section 3.4. The problem of the optimal tuning of Q can then be summarized as follows:

(8) $$\begin{array}{ccc} \mathrm{Find}\;\sigma_{opt}^{2*}\in \; & \; & \min_{\sigma_{opt}^{2}\in R^{11}}\sum_{j=1}^{N_{\theta }}\sum_{k=1}^{N}{\left(q_{SS_{jk}}-q_{EKF_{jk}}\right)}^{2}\\ \mathrm{subject}\;\mathrm{to} & & \\ \; & \; & \sigma_{opt}^{2-}\leq \sigma_{opt}^{2}\leq \sigma_{opt}^{2+} \end{array}$$

where *N* is the number of samples of a considered task, and $\sigma_{opt}^{2-}$ = 1 × 10^−3^ and $\sigma_{opt}^{2+}$ = 1 × 10^2^ are the lower and upper boundaries on the Q elements. This problem was solved using a classical trust-region-reflective optimization algorithm [35].

### 3.5. Performance Analysis

The ability of the proposed CEKF and of its optimal covariance matrix tuning to accurately estimate the joint kinematics was assessed first by calculating the RMSD and the Pearson correlation coefficient (CC) between the estimated and reference joint angles. These calculations were performed for joints that were of interest for each task. Moreover, in order to assess the distribution of the differences between the estimated and reference joint trajectories, a statistical parametric mapping (SPM) analysis was devised [36]. SPM was developed to evaluate inferences regarding the topological features of statistical processes that are continuous functions of space and time. Statistical differences among continuous curves can be analyzed without reducing the dimensions of the test to summarize metrics such as mean, median, maximum or minimum values. The SPM method is used to analyze the performance of the CEKF when the elements of Q are optimized, as in Section 3.4.2, as opposed to when they are estimated based on a classical method from the literature [34].

In order to determine the influence of this tuning, the absolute difference between the estimated and reference joint angles was calculated and compared with a 1D paired *t*-test (α=0.05). Tests were carried out with the open-source package SPM1D for MATLAB [37], which generated the map of t-values SPM{t}, the t* boundaries and the areas for relevant p-values.

## 4. Results and Discussion

**Figure 4:**
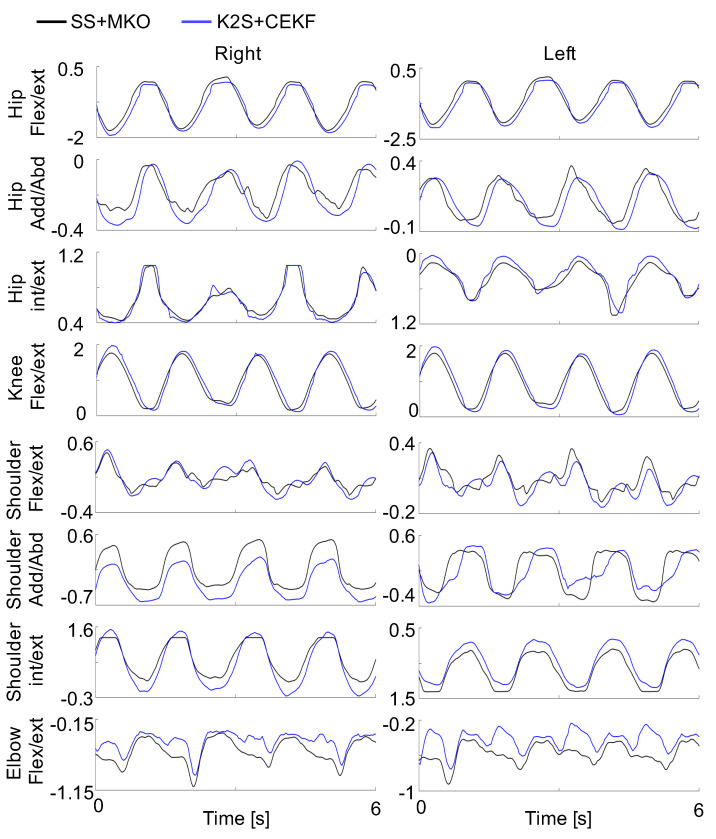
Representative comparison of joint trajectories estimated using the K2S and the proposed constrained extended Kalman filter (CEKF) (blue) and the SS and multibody kinetic optimization (MKO) (black) during the squat task. Flex/ext, int/ext and add/abd represent flexion/extension, internal/external rotation and adduction/abduction, respectively.

Figure 4 shows a representative comparison between the joint angles estimated with the proposed, optimally tuned CEKF and those obtained from the reference SS and MKO for a randomly chosen subject over four repetitions of the squat task. The corresponding RMSD is 11.0±7.8${}^{\circ }$ and the CC is 0.85±0.19. The ability of the CEKF to constrain the joint kinematics is clearly visible for the hip internal/external rotations.

The average RMSD and CC of all tasks and angles of interest are 9.7${}^{\circ }$ and 0.8 when using the optimally tuned CEKF, respectively. When the CEKF covariance matrices are tuned from measured data only (see Section 3.4), these values are much larger (RMSD =16.4${}^{\circ }$, CC = 0.70). Consequently, only the results when CEKF is optimally tuned are presented in this section. RMSD and CC are calculated for all joints of interest and all trials and are reported separately for each task in Table 1.

**Figure 5:**
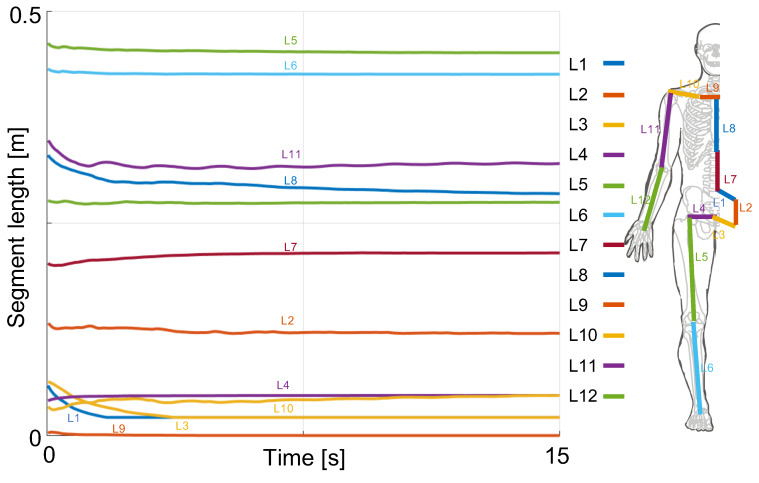
Representative evolution of the estimation of the segment lengths corresponding to the model described in Section 3.1. The segment lengths converge toward a constant fixed realistic value.

Figure 5 shows that, as expected, by setting the corresponding elements of Q to zero, the segment lengths converge toward a constant value. This has the effect of constraining the joint kinematics solution, similar to when state of-the-art MKO is used offline [3], and in contrast to when no model is used [38]. Moreover, one can see that the segment lengths are always positive and thus are physically consistent.

For all observed tasks, the internal/external rotations of the hips and of the shoulder display the largest RMSD. This might be explained by the fact that, with the selected input data (i.e., joint center positions), there are, in theory, an infinite number of solutions to solve the inverse kinematics for these angles. The segment orientation estimate, provided as quaternions by the K2S, is not reliable enough to be used as additional input data. With one degree-of-freedom at the knees and elbows, and using the previous state in the CEKF, a reasonable solution can be found unless flexion remains close to 0 for these joints. Adding the ankle and wrist joints into the kinematic model and the fingers and feet JCPs into the input data would have also negatively impacted the results since their reliability is questionable.

A large RMSD can also be observed for the shoulder flexion/extension, especially during the execution of the squat exercise. This is due to the fact that the arm and shoulder elevations are shared between the clavicle and the shoulder flexion/extension. In fact, unlike the reference system, which relies on a redundant set of retro-reflective markers, the proposed CEKF cannot dissociate the contribution of each joint. Moreover, the shoulder JCP only is provided by the K2S. Still, the clavicle segment is necessary due to the nature of the investigated tasks and potential future tasks involving high arm elevations. Nevertheless, as shown by the good average CC and by Figure 4, the shape of the joint angle is relatively well preserved. Finally, a recent review has reported the typical errors for the model-derived glenohumeral rotations [3] and showed that errors are maximal for internal/external rotations and reach 10${}^{\circ }$ compared to the ground truth (i.e., bone pins or fluoroscopy). This is close to the result obtained in this study, with an average of 14${}^{\circ }$ RMSD and 0.80 CC for all data collected considering SS with MKO as the reference.

For the lower limbs, tasks 1 and 2 exhibited similar results despite the fact that the ankle JCP is moving in task 2. The highest RMSD was obtained for the hip joints $\theta_{4}$, $\theta_{6}$, $\theta_{8}$ and $\theta_{10}$. Interestingly, knee joint angles were well estimated with an average RMSD of 7${}^{\circ }$ and an excellent CC of 0.985. As exemplified in Figure 4, the difference in the knee joint angle essentially arises from the amplitude, which is systematically reduced when using K2S data.

The internal/external rotation of the hip showed worse results in task 2 (above 17${}^{\circ }$) than Task 1 (below 13${}^{\circ }$). The difference in the internal/external rotation between these two trials can be explained by the position of the ankles, which are fixed in squat exercises but not in the stepping task. Moreover, these joints can be compared to the shoulder and suffer from the same type of error stated above.

In the literature about K2S, the best results were obtained for the trunk with a calculated RMSD and CC of 3.7${}^{\circ }$ and 0.83, respectively. Consequently, as expected, tasks 3 and 4 presented very satisfactory results for trunk and hip joint angle estimates, with an average RMSD of 3.6${}^{\circ }$ and 5.5${}^{\circ }$ for task 3 and 3.5${}^{\circ }$ and 5.5 ${}^{\circ }$ for task 4, respectively.

### Q Matrix Estimation

**Figure 6:**
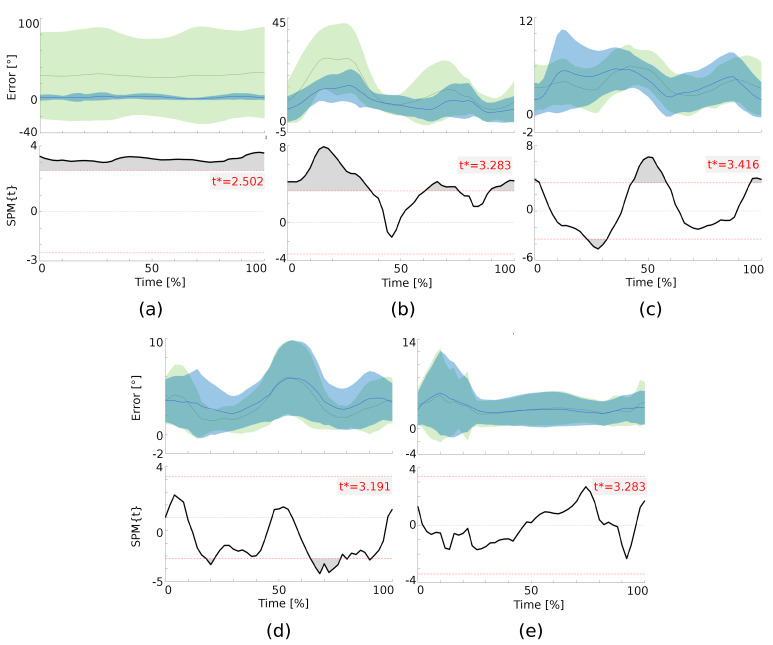
Representative results obtained with the paired t-test statistical parametric mapping (SPM). The top figures show descriptive statistics for each motion (Mean ±1 SD error cloud from between-subject variability) when the CEKF’s covariance matrices are optimized (green) and when classical methods are used to tune them (blue). Bottom figures show the most frequent inferences. The thick black line depicts the test statistic continuum or SPM{t}. The red horizontal dashed lines illustrate the critical t* based on α=0.05. All areas outside the dashed red lines (grey) represent a p-value inferior to 5%. (**a**) is the angle $\theta_{14}$ for task 1, (**b**) the angle $\theta_{4}$ of task 1, (**c**) the angle $\theta_{5}$ of task 4, (**d**) the angle $\theta_{5}$ of task 2, and (**e**) is the angle $\theta_{9}$ of task 3

Since each task contains ten repetitions, a total of 40 different 1D paired *t*-tests were performed on angles of interest. Representative examples of these tests are shown in Figure 6. The upper figures show descriptive statistics while the bottom ones present results of the *t*-test obtained with the SPM method. A large number of tests (33/40) showed significant differences in favor of the method optimizing the CEKF’s covariance matrices. This means that, when optimizing the CEKF covariance matrices, the estimated joint angles are significantly closer to the reference estimates than when using the classical tuning method of the CEKF covariance matrices. However, the evolution of these differences during the motion cycle should be assessed. The use of the SPM analysis allows us to show when the differences are significant during motion. Figure 6a, obtained for the flexion/extension of the right shoulder ($\theta_{14}$) during task 1, shows the only case in which a significant difference between the optimally and data driven tuning methods was observed during the whole motion. Figure 6b, obtained for the right hip flexion/extension ($\theta_{4}$) during task 1, illustrates the 22 cases over 33 when significant differences for more than 60% of the motions are observed. The most significant differences are consistently observable at the beginning and at the end of the motion.

Four tests illustrate significant differences in favor of both tuning methods. Figure 6c shows this for the right hip abduction/adduction ($\theta_{5}$) of task 4. This test obtains a *p*-value = 0.043 at the beginning of the motion in favor of the optimization approach (positive supra-threshold), a *p*-value = 0.003 at 25% of the motion cycle in favor of the classical tuning method, a *p*-value < 0.0001 at 50% and a *p*-value = 0.021 at the end of the motion. Similar results are found for the first and third tasks, for the joint angles $\theta_{15}$, $\theta_{21}$ and $\theta_{5}$.

Figure 6d, obtained for the right hip abduction/adduction ($\theta_{5}$) of task 2, presents the only test in which the classical method to tune the CEKF covariance matrices gives a better result than when an optimization process is used. Consequently, the supra-threshold clusters are negative, but with a small amplitude and only for some limited phases of the motion. Two tests show no significant differences between the two CEKF covariance matrices tuning methods. Figure 6e, obtained for the left hip abduction/adduction ($\theta_{9}$) of task 3, exemplifies this observation. In this figure, it can be seen that the SPM{t} curve is always located between the horizontal dashed lines indicating the critical t* (α=0.05). It is interesting to note that both non-significant and significant outcomes of *t*-test SPM in favor of the classical tuning method presented a small RMSD (about 5${}^{\circ }$). This analysis shows that the method is indeed sensitive to the definition of Q and R. In the present study, the covariance matrices, whether optimized or not, are determined based on the data coming from the SS. This impedes the current use of the method as a stand-alone approach; i.e., without referring to SS with MKO to adjust these parameters for each task.

## 5. Conclusions

The purpose of the present study was to show the possibility of improving the accuracy of joint angle estimates from data provided by a Kinect sensor 2 using a constrained biomechanical model. The covariance matrices of the proposed constrained EKF were optimally tuned for each investigated task from the data of one subject. When applying the identified parameters to the other subjects, the accuracy analysis showed a relatively good estimate of joint angles with the exception of the internal/external rotations of hip and shoulder joints. This shows that the proposed tuning method is task-dependent and thus that the same values of Q and R can be used for any other healthy individual. If a new exercise is investigated or if the K2S location is largely modified, then the optimal tuning described in Equation (1) should be performed again.

Additionally, the tuning of Q allows us to obtain constant segment lengths that are robust to the disturbance of the K2S JCP estimates. Figure 5 shows the good convergence to each of the segment lengths while realistic positive values and realistic segment proportions are maintained. In addition to always having realistic segment lengths, the physical constraints described in Equation (7) ensure that the joint angle estimates are physically feasible, as shown in Figure 4. As supported by the literature, having a physically consistent estimate of segment lengths and joint angles is of crucial importance to conveying meaningful information in both rehabilitation and training settings. The proposed approach can be easily transferred to daily practice outside the laboratory, and its use requires no special skills. A limitation of the proposed approach is that it does not rely on the full set of information provided by the K2S. Analyses are only performed with 15 of the 24 JCPs estimated by the K2S, and the quaternions used to represent the segment orientations are not included. In fact, large variations and discontinuities in the quaternion data were observed during the investigated tasks. Similarly, ankle angles are not estimated because of the noisy feet positions obtained by the skeleton extractor of the K2S. However, thanks to the simple formulation of constraints in the proposed CEKF, it is possible to get the ankle angles by adding constraints on feet positions. A state estimator monitoring the height of the ankle and foot JCPs would have to be added to the proposed CEKF. Affordable balance sensors (e.g., a Wii Balance Board) could provide more information on foot position and the dynamic behavior of the locomotor system. Including dynamics would improve the consistency of joint kinematics [21]; however, its inclusion into a CEKF would have a large computational cost that might not meet the requirement of a low-cost device which could be used for in-home rehabilitation.

If lifting weights were to be used in the hands and/or attached to a specific segment, the accuracy of the KS2 JCP estimate should be assessed. The Microsoft SDK JCP estimation method, and thus its robustness to modifications, is undisclosed. Thus, any new modification of the experimental setup would, in theory, require a new calibration procedure with a reference motion capture system. This is the main limitation of the proposed approach.

Although it was not investigated in this study, the spatio-temporal parameters of gait, which are of great interest to the clinical community, could be estimated with the proposed approach. Indeed, variables such as gait velocity, pelvis oscillation amplitude or step lengths can be reconstructed from joint kinematics and a correct estimate of segment lengths. However, this would have to be done on a treadmill; i.e., keeping the patient–K2S distance within strict limits.

Finally, the proposed CEKF method and its tuning could be used to ensure physical consistency with the new generation of Kinect sensor. This new sensor is expected to have a largely improved accuracy that might allow the use of additional information (hand and foot positions and segment orientation) that are ignored in this study. One advantage of the proposed solution is that a change of the sensor will only impact the algorithm tuning. Covariance matrices will need to be optimized for each task with a reference SS and MKO. However, unlike algorithms based on machine learning which are sensor-dependent and necessitate a large amount of data, only one subject per task could be used to tune the parameters of the proposed method.

## Figures and Tables

**Table 1:** Joint angle estimation obtained using the optimal tuning of Q. Accuracy for angles of interest have been reported as mean ± SD over all the analyzed trials. RMSD: root mean square difference; CC: correlation coefficient.

**Task 1**									
		$\theta_{4}$	$\theta_{5}$	$\theta_{6}$	$\theta_{7}$	$\theta_{8}$	$\theta_{9}$	$\theta_{10}$	$\theta_{11}$
	RMSD (${}^{\circ }$)	11.3 ± 2.6	3.0 ± 1.1	8.6 ± 3.5	6.5 ± 1.1	12.2 ± 3.3	3.9 ± 2.3	13.4 ± 7.0	7.2 ± 2.2
**Lower Body**	CC	0.98 ± 0.01	0.85 ± 0.06	0.93 ± 0.05	0.99 ± 0.00	0.98 ± 0.01	0.85 ± 0.09	0.76 ± 0.35	0.98 ± 0.01
		$\theta_{14}$	$\theta_{15}$	$\theta_{16}$	$\theta_{17}$	$\theta_{19}$	$\theta_{20}$	$\theta_{21}$	$\theta_{22}$
	RMSD	7.5 ± 3.1	25.6 ± 6.4	15.3 ± 6.0	9.8 ± 2.4	8.3 ± 5.2	20.0 ± 5.3	17.5 ± 4.7	10.2 ± 2.1
**Upper Body**	CC	0.78 ± 0.01	0.78 ± 0.15	0.94 ± 0.05	0.66 ± 0.24	0.80 ± 0.12	0.70 ± 0.18	0.93 ± 0.06	0.62 ± 0.2
**Task 2**									
		$\theta_{4}$	$\theta_{5}$	$\theta_{6}$	$\theta_{7}$	$\theta_{8}$	$\theta_{9}$	$\theta_{10}$	$\theta_{11}$
	RMSD	8.1 ± 3.5	4.0 ± 2.0	17.9 ± 10.1	8.3 ± 5.4	9.7 ± 5.4	3.7 ± 2.5	20.4 ± 12.9	8.2 ± 8.1
**Lower Body**	CC	0.98 ± 0.03	0.54 ± 0.33	0.81 ± 0.19	0.98 ± 0.03	0.97 ± 0.06	0.76 ± 0.16	0.69 ± 0.26	0.97 ± 0.05
**Task 3**									
		$\theta_{4}$	$\theta_{5}$	$\theta_{8}$	$\theta_{9}$	$\theta_{12}$			
	RMSD	9.1 ± 1.9	4.7 ± 2.6	9.7 ± 2.3	4.3 ± 2.5	6.7 ± 1.9			
**Lower Body + Trunk**	CC	0.97 ± 0.04	0.86 ± 0.15	0.97 ± 0.04	0.86 ± 0.19	0.79 ± 0.31			
**Task 4**									
		$\theta_{4}$	$\theta_{5}$	$\theta_{8}$	$\theta_{9}$	$\theta_{12}$			
	RMSD	8.7 ± 3.8	5.6 ± 1.6	8.9 ± 3.6	5.0 ± 1.2	5.4 ± 2.5			
**Lower Body + Trunk**	CC	0.88 ± 0.09	0.98 ± 0.01	0.85 ± 0.10	0.98 ± 0.01	0.77 ± 0.32			

## References

[1] Brunnekreef J.J., van Uden C.J., van Moorsel S., Kooloos J.G. (2005). Reliability of videotaped observational gait analysis in patients with orthopedic impairments. BMC Musculoskelet. Disord..

[2] Lavernia C., D’Apuzzo M., Rossi M.D., Lee D. (2008). Accuracy of Knee Range of Motion Assessment after Total Knee Arthroplasty. J. Arthroplast..

[3] Begon M., Andersen M.S., Dumas R. (2018). Multibody Kinematics Optimization for the Estimation of Upper and Lower Limb Human Joint Kinematics: A Systematized Methodological Review. J. Biomech. Eng..

[4] Bonnechère B., Jansen B., Omelina L., Van Sint Jan S. (2016). The use of commercial video games in rehabilitation: A systematic review. Int. J. Rehabil. Res..

[5] López-Nava I.H., Muñoz-Meléndez A. (2016). Wearable Inertial Sensors for Human Motion Analysis: A Review. IEEE Sens. J..

[6] Moeslund T.B., Granum E. (2001). A Survey of Computer Vision-Based Human Motion Capture. Comput. Vis. Image Underst..

[7] Chen L., Wei H., Ferryman J. (2013). A survey of human motion analysis using depth imagery. Pattern Recognit. Lett..

[8] Cao Z., Hidalgo G., Simon T., Wei S.E., Sheikh Y. (2018). OpenPose: Realtime Multi-Person 2D Pose Estimation using Part Affinity Fields. arXiv.

[9] Li Z., Sedlar J., Carpentier J., Laptev I., Mansard N., Sivic J. Estimating 3D Motion and Forces of Person-Object Interactions From Monocular Video. Proceedings of the 2019 IEEE/CVF Conference on Computer Vision and Pattern Recognition (CVPR).

[10] Da Gama A., Fallavollita P., Teichrieb V., Navab N. (2015). Motor Rehabilitation Using Kinect: A Systematic Review. Games Health J..

[11] Wang Q., Kurillo G., Ofli F., Bajcsy R. Evaluation of Pose Tracking Accuracy in the First and Second Generations of Microsoft Kinect. Proceedings of the International Conference on Healthcare Informatics.

[12] Naeemabadi M., Dinesen B., Andersen O.K., Najafi S., Hansen J. Evaluating Accuracy and Usability of Microsoft Kinect Sensors and Wearable Sensor for Tele Knee Rehabilitation after Knee Operation. https://www.scitepress.org/papers/2018/65782/65782.pdf.

[13] Wochatz M., Tilgner N., Mueller S., Rabe S., Eichler S., John M., Völler H., Mayer F. (2019). Reliability and validity of the Kinect V2 for the assessment of lower extremity rehabilitation exercises. Gait Posture.

[14] Galna B., Barry G., Jackson D., Mhiripiri D., Olivier P., Rochester L. (2014). Accuracy of the Microsoft Kinect sensor for measuring movement in people with Parkinson’s disease. Gait Posture.

[15] Otte K., Kayser B., Mansow-Model S., Verrel J., Paul F., Brandt A.U., Schmitz-Hübsch T. (2016). Accuracy and Reliability of the Kinect Version 2 for Clinical Measurement of Motor Function. PLoS ONE.

[16] Bonnechère B., Sholukha V., Omelina L., Van Sint S., Jansen B. (2018). 3D Analysis of Upper Limbs Motion during Rehabilitation Exercises Using the KinectTM Sensor: Development, Laboratory Validation and Clinical Application. Sensors.

[17] Kuster R.P., Heinlein B., Bauer C.M., Graf E.S. (2016). Accuracy of KinectOne to quantify kinematics of the upper body. Gait Posture.

[18] Han F., Reily B., Hoff W., Zhang H. (2017). Space-time representation of people based on 3D skeletal data: A review. Comput. Vis. Image Underst..

[19] Plantard P., Shum H.P.H., Multon F. (2017). Filtered pose graph for efficient kinect pose reconstruction. Multimed. Tools Appl..

[20] Kim Y., Baek S., Bae B.C. (2017). Motion Capture of the Human Body Using Multiple Depth Sensors. ETRI J..

[21] Skals S., Rasmussen K.P., Bendtsen K.M., Yang J., Andersen M.S. (2017). A musculoskeletal model driven by dual Microsoft Kinect Sensor data. Multibody Syst. Dyn..

[22] Chen C., Jafari R., Kehtarnavaz N. (2017). A survey of depth and inertial sensor fusion for human action recognition. Multimed. Tools Appl..

[23] Feng S., Murray-Smith R. Fusing Kinect sensor and inertial sensors with multi-rate Kalman filter. Proceedings of the IET Conference on Data Fusion and Target Tracking: Algorithms and Applications (DF TT 2014).

[24] Du Y.C., Shih C.B., Fan S.C., Lin H.T., Chen P.J. (2018). An IMU-compensated skeletal tracking system using Kinect for the upper limb. Microsyst. Technol..

[25] Tripathy S.R., Chakravarty K., Sinha A. Constrained Particle Filter for Improving Kinect Based Measurements. Proceedings of the 26th European Signal Processing Conference (EUSIPCO).

[26] Shu J., Hamano F., Angus J. (2014). Application of extended Kalman filter for improving the accuracy and smoothness of Kinect skeleton-joint estimates. J. Eng. Math..

[27] Khalil W., Creusot D. (1997). SYMORO+: A system for the symbolic modelling of robots. Robotica.

[28] Wu G., van der Helm F.C.T., (DirkJan) Veeger H.E.J., Makhsous M., Van Roy P., Anglin C., Nagels J., Karduna A.R., McQuade K., Wang X. (2005). International Society of Biomechanics, Standardization and Terminology Committee. ISB recommendation on definitions of joint coordinate systems of various joints for the reporting of human joint motion—Part II: Shoulder, elbow, wrist and hand. J. Biomech..

[29] Gupta N., Hauser R. (2017). Kalman Filtering with Equality and Inequality State Constraints. arXiv.

[30] Yang L., Zhang L., Dong H., Alelaiwi A., Saddik A.E. (2015). Evaluating and Improving the Depth Accuracy of Kinect for Windows v2. IEEE Sens. J..

[31] Naeemabadi M., Dinesen B., Andersen O.K., Hansen J. (2019). Influence of a Marker-Based Motion Capture System on the Performance of Microsoft Kinect v2 Skeleton Algorithm. IEEE Sens. J..

[32] Davis R.B., Õunpuu S., Tyburski D., Gage J.R. (1991). A gait analysis data collection and reduction technique. Hum. Mov. Sci..

[33] Cerveri P., Rabuffetti M., Pedotti A., Ferrigno G. (2003). Real-time human motion estimation using biomechanical models and non-linear state-space filters. Med. Biol. Eng. Comput..

[34] De Groote F., De Laet T., Jonkers I., De Schutter J. (2008). Kalman smoothing improves the estimation of joint kinematics and kinetics in marker-based human gait analysis. J. Biomech..

[35] Coleman T., Li Y. (1996). An Interior Trust Region Approach for Nonlinear Minimization Subject to Bounds. Siam J. Optim..

[36] Friston K.J., Ashburner J.T., Kiebel S.J., Nichols T.E., Penny W.D. (2011). Statistical Parametric Mapping: The Analysis of Functional Brain Images.

[37] http://www.spm1d.org/.

[38] Donati M., Camomilla V., Vannozzi G., Cappozzo A. (2008). Anatomical frame identification and reconstruction for repeatable lower limb joint kinematics estimates. J. Biomech..

